# Neural network-based estimation of biomechanical vocal fold parameters

**DOI:** 10.3389/fphys.2024.1282574

**Published:** 2024-02-21

**Authors:** Jonas Donhauser, Bogac Tur, Michael Döllinger

**Affiliations:** Division of Phoniatrics and Pediatric Audiology, Department of Otorhinolaryngology, Head and Neck Surgery, University Hospital Erlangen, Friedrich-Alexander University Erlangen-Nürnberg, Erlangen, Germany

**Keywords:** convolutional recurrent neural network, high-speed video, mass–spring–damper system, vocal fold dynamics, voice physiology

## Abstract

Vocal fold (VF) vibrations are the primary source of human phonation. High-speed video (HSV) endoscopy enables the computation of descriptive VF parameters for assessment of physiological properties of laryngeal dynamics, i.e., the vibration of the VFs. However, underlying biomechanical factors responsible for physiological and disordered VF vibrations cannot be accessed. In contrast, physically based numerical VF models reveal insights into the organ’s oscillations, which remain inaccessible through endoscopy. To estimate biomechanical properties, previous research has fitted subglottal pressure-driven mass–spring–damper systems, as inverse problem to the HSV-recorded VF trajectories, by global optimization of the numerical model. A neural network trained on the numerical model may be used as a substitute for computationally expensive optimization, yielding a fast evaluating surrogate of the biomechanical inverse problem. This paper proposes a convolutional recurrent neural network (CRNN)-based architecture trained on regression of a physiological-based biomechanical six-mass model (6 MM). To compare with previous research, the underlying biomechanical factor “subglottal pressure” prediction was tested against 288 HSV *ex vivo* porcine recordings. The contributions of this work are two-fold: first, the presented CRNN with the 6 MM handles multiple trajectories along the VFs, which allows for investigations on local changes in VF characteristics. Second, the network was trained to reproduce further important biomechanical model parameters like VF mass and stiffness on synthetic data. Unlike in a previous work, the network in this study is therefore an entire surrogate of the inverse problem, which allowed for explicit computation of the fitted model using our approach. The presented approach achieves a best-case mean absolute error (MAE) of 133 Pa (13.9%) in subglottal pressure prediction with 76.6% correlation on experimental data and a re-estimated fundamental frequency MAE of 15.9 Hz (9.9%). In-detail training analysis revealed subglottal pressure as the most learnable parameter. With the physiological-based model design and advances in fast parameter prediction, this work is a next step in biomechanical VF model fitting and the estimation of laryngeal kinematics.

## 1 Introduction

**FIGURE 1:**
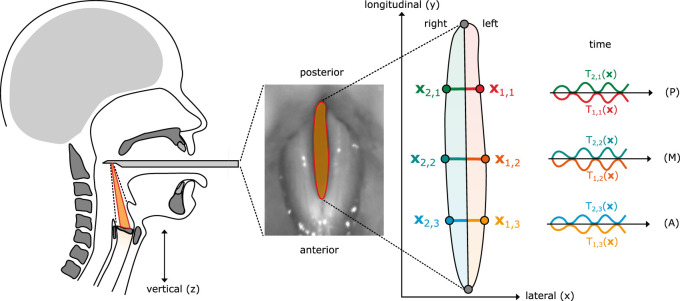
Schematic drawing for high-speed video (HSV) endoscopy of the vocal folds (VFs). By frame-wise segmentation, the glottal area between the two VFs can be extracted. Trajectories (A = anterior, M = medial, and P = posterior) are the time-wise deflection of the VFs to the glottal midline (Lohscheller et al., 2013).

Phonation is the engine behind daily human communication. Whether at work, in school, or for casual conversations, having voice problems hinders social interaction, which may lead to depression and other mental health problems (Nerriere et al., 2009). For the diagnostics of an affected patient, nasal and oral endoscopy is often used by physicians to inspect potentially irregular oscillating vocal folds (VFs). While the most popular recording technique is still stroboscopy (Fukahori et al., 2016), indirectly recording the organs’ motion by periodic imaging through light strobes, modern high-speed video (HSV) endoscopy systems are able to precisely record the motion (Figure 1) at more than 4,000 frames per second (FPS), enabling detailed research on VF motion (Kunduk et al., 2010; Schutzenberger et al., 2016).

**FIGURE 2:**
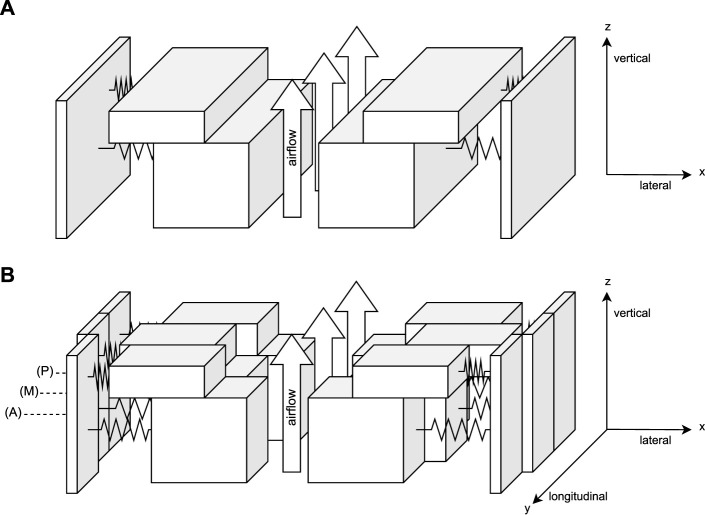
Schematic vocal fold (VF) models. Each VF is represented by multiple coupled masses, and the coupling springs between adjacent masses are not shown. The system is oscillated by tracheal airflow **(A)** Two-mass model by Ishizaka and Flanagan (1972). **(B)** Six-mass model by Schwarz et al. (2008).

Not only disordered voices can be identified through HSV (Inwald et al., 2011), but also visually inaccessible quantities like subglottal pressure, which is comparably increased in dysphonic patients (Ketelslagers et al., 2007; Giovanni et al., 2000), and tissue characteristics (Moore and Thibeault, 2012; Björklund and Sundberg, 2016) are crucial for voice production. To get further insights, biomechanical mass–spring–damper (MSD) systems are used to simulate physiologically based VF motion with a given set of model parameters. Even though being motivated as a substitute to inaccessible anatomical tissue properties, the parameter’s use should be rather seen as a kinematic VF representation, rather than in being an exact estimate for anatomical mass and stiffness. One of the earliest biomechanical VF models is the two-mass model (2 MM) developed by Ishizaka and Flanagan (1972), based on two spring coupled point masses per side, oscillated through a Bernoulli airflow-based driving force. Steinecke and Herzel (1995) simplified the model to its commonly used form, for which coherences between parameter adjustments, left–right asymmetries, and clinical observations have been shown (Story and Titze, 1995; Fraile et al., 2012). While the simple model successfully captures many important phonatory characteristics, a major shortcoming is the oversimplification of the tissue as a linear spring (Gray et al., 2000; Zhang K. et al., 2006a; Döllinger et al., 2011), which was resolved by adding a cubic term to the spring response (Fulcher et al., 2006; Gómez et al., 2018). A limitation is the absence of longitudinal coordinates in the 2 MM, such that the 2 MM prohibits meaningful reconstruction of the glottal area. By dividing the 2 MM into three spring-interconnected longitudinal sections as shown in Figure 2, the six-mass model (6 MM) by Schwarz et al. (2008) was obtained. Through this augmentation, to multiple tracking points in the longitudinal direction, localized adaption of tissue biomechanics is enabled. By this, the 6 MM can account for polyps, and match local differences in VF dynamics, which are of particular interest to analyze functional dysphonia with disordered oscillations. Anterior- to posterior-wise differing VF geometry positions can furthermore account for an increased glottal gap, which is prevalent in women (Cielo et al., 2019). By further increasing spring mesh resolution, which is, e.g., preferable for medial VF surface analysis, multi-mass models were obtained (Yang et al., 2010). To better account for differing tissue layers, Story and Titze (1995) extended the classical 2 MM to a so-called body-cover model by adding an extra ”body” mass in the lateral direction. None of the presented models consider acoustic coupling effects due to vocal tract interactions, which increasedly impacts phonation at higher fundamental frequencies and causes phenomena like frequency jumps and subharmonics (Zhang Z. et al., 2006b; Titze et al., 2008; Lucero et al., 2012). Systematic model reviews can further be found in Birkholz (2011) and Erath et al. (2013).

The availability of precise HSV recordings, paired with increasing computational power, enabled the adaption of simple numerical models to recorded trajectories. To reveal visually inaccessible biomechanical properties from VF oscillation, the computed trajectories of an MSD model were automatically fitted to experimentally recorded trajectories by optimization (Döllinger et al., 2002; Schwarz et al., 2008). Asymmetries in the parameters of the fitted 2 MM have been shown to be indicative of disordered voice and can be used to identify pathological cases (Schwarz et al., 2006; Wurzbacher et al., 2006; Zhang et al., 2018). Furthermore, coherence between longitudinal variation of optimized 6 MM parameters and different pathologies has been found (Schwarz et al., 2008).

In general, the optimization problem’s complexity vastly depends on its search space dimension (Long et al., 2019), i.e., the non-fixed model parameters. The complexity scales with the evaluation time underlying differential problems, as it is required to solve the models’ differential equations not once, but many times, as re-evaluation of the model is required. For the simplistic 2 MM, a differential evolution approach for a 14-degrees-of-freedom (DOF)-based optimization by Gómez et al. (2018) was reported to need more than 100 k model evaluations to achieve convergence. As more sophisticated VF models, like finite volume-based models, require hours to days per evaluation (Falk et al., 2021), optimization of complex models is computationally demanding. To avoid repeated computational effort for optimization, it is apparent to train a neural network (NN) as a fast evaluating surrogate, which requires computing a large synthetic instance–solution dataset beforehand and only once (Gómez et al., 2019). The idea is to learn complex relations between samples by iteratively adapting trainable weights, such that the surrogate NN generalizes to the core problem (Nielsen, 2015).

For time-series problems (Foumani et al., 2023), such as fitting VF model parameters to given trajectories, recurrent neural networks (RNNs) (Rumelhart et al., 1986) are commonly used, as they do not require fixed length input (Fawaz et al., 2018). A popular RNN variant for learning long sequences is long short-term memory (Hochreiter and Schmidhuber, 1997), which was trained by Gómez et al. (2019) on subglottal pressure prediction, using a hidden size of 128. A drawback of RNNs is that they are comparably slow in training (Fawaz et al., 2018), and long sequences tend to cause exploding and vanishing gradients (Bengio et al., 1994; Pascanu et al., 2012). The key improvement in convolutional recurrent neural networks (CRNNs) (Zhou et al., 2015) over plain RNN-based architectures is the incorporation of convolution layers as initial feature compression before the data are processed by a comparably slow RNN core unit. To fix long-term sequence problems, attention-mechanisms (Bahdanau et al., 2014; Brauwers and Frasincar, 2022) were introduced.

In addition to trajectory-based pressure estimation, feedforward neural networks (FFNNs) are also investigated as an audio-feature-based geometry predictor of a VF body-cover model (Zhang, 2020). A similar approach was used by Ibarra et al. (2021) for pressure prediction through neck-surface accelerometer-obtained features.

We contribute to the state of the art by providing a specially designed neural network (NN) for the trajectory-based parameter prediction for a 6 MM. In particular, our method is not solely trained on pressure estimation but on full parameter inversion, in analogy to classical optimization approaches (Döllinger et al., 2002; Gómez et al., 2018), through which we state insights on NN-based learnability of 6 MM key parameters. For synthetic data generation, we present a gamma distribution-based rest position sampling strategy using copulas (Nelsen, 2006).

## 2 Methods

In this section, the biomechanical 6 MM for VF modeling by Schwarz et al. (2008) is introduced. The corresponding inverse problem is formally stated as a differential equation-constrained optimization problem. To overcome the comparably large computational effort of directly solving the optimization, a convolutional recurrent neural network (CRNN) is introduced as a surrogate for the inverse problem.

### 2.1 One dimensional six-mass model

The 6 MM (Schwarz et al., 2008) represents each VF as a longitudinal–vertical (cf. Figure 2)-oriented 3 × 2 mesh of spring interconnected masses. We simplified the original 6 MM, to a dimensionality-reduced 1D variant, exploiting that major VF motion happens in the medio-lateral direction (Döllinger et al., 2005; Döllinger et al., 2016). Like in the 2 MM (Steinecke and Herzel, 1995), VF motion is restricted to a single DOF, requiring the state of the differential equation to be solely computed in lateral coordinates.

To ease notation, we introduce masses $m\in R_{>0}^{2\times 3\times 2}$ with their associated lateral positions $x\in R^{2\times 3\times 2}$ as tensors. Temporal derivatives of first and second order are denoted as $\dot{x}$ and $\ddot{x}$ respectively. In addition to the spring mesh forces in the longitudinal and vertical direction, given by ***F*** ^*v*^ and ***F*** ^*l*^, respectively, the masses are impacted by three lateral directed forces. The anchor force ***F*** ^*a*^ draws the masses to specified lateral rest positions $x^{r}\in R^{2\times 3\times 2}$ . The collision force ***F*** ^*c*^ is a push back force that handles VF overlapping, and last, the Bernoulli airflow pressure-based driving force ***F*** ^*d*^, pushing the glottis into open state, depends on the level of closure. Formally, each force tensor $F^{•}\in {\left(R^{3}\right)}^{2\times 3\times 2}$ is a grid of three-dimensional force vectors that act on the masses, and • ∈ {*a*, *v*, *c*, *l*} is used as placeholder. Except for the driving force ***F*** ^*d*^, each tensor component is based on damped linear springs (Eq. 1) with vectorial deflection $s≔{\left(x,y,z\right)}^{T}\in R^{3}$

$$F_{k_{•},r_{•}}\left(s,\ell_{•}\right)≔-k_{•}\;\left(\|s\|-\ell_{•}\right)\left(\frac{s}{\left\|s\right\|}\right)+r_{•}{\left(\frac{s}{\left\|s\right\|}\right)}^{T}\dot{s}\left(\frac{s}{\left\|s\right\|}\right),$$ (1)

with associated scalar-free elongation *ℓ*_•_, stiffness *k*_•_, and damping factor *r*_•_. An explicit formulation of all forces can be found in Supplementary Material. Friction is solely assumed for the anchor force, i.e., *r*_*l*_, *r*_*v*_, *r*_*c*_ = 0, and *ℓ*_*a*_, *ℓ*_*c*_ = 0 is assumed for lateral springs. Non-linear springs are assumed to increase the realism of Eq. 1: for the anchor force ***F*** ^*a*^ extended by a cubic term, i.e., multiplied by (1 + *ηx* ^2^) with *η* = 100 (Fulcher et al., 2006). For the vertical and longitudinal coupling ***F*** ^*v*^, ***F*** ^*l*^, we used $k_{•}\;\sigma \left(x\right)\left(\sqrt{x^{2}+\ell_{•}^{2}}-\ell_{•}\right)$ as the lateral spring response, which is discussed in Section 4.4, and *σ*(*x*) denotes the sigmoid function. The stiffness *k*_*l*_ of a spring between two masses is set proportionally (factor *ξ*_*l*_ = 0.2) to the summed adjacent anchor stiffnesses *k*_*a*_, and likewise, the collision spring stiffness is set to *k*_*c*_ = *k*_*a*_ ⋅ *ξ*_*c*_ with factor *ξ*_*c*_ = 1. In summary, lateral motion can be stated as a second-order ordinary differential equation (ODE) (Eq. 2):

$$F\left(x,\dot{x}\right)≔{\left[F^{a}+F^{v}+F^{c}+F^{d}+F^{l}\right]}_{x}=m⊙\ddot{x},$$ (2)

where [⋅]_*x*_ denotes lateral component selection, and component-wise multiplication is denoted as ⊙.

In analogy to the HSV camera perspective, the model’s trajectories *T*(***x***) are defined as the minimal distance of each vertical mass pair to the glottal midline in a lateral–longitudinal projection (cf. Figure 1). Given a set of experimentally recorded trajectories *T* ^exp^, the most plausible model parameters (Eq. 3) can be defined as the ones which best reproduce the observed trajectories. The computed trajectories *T*(***x***) should therefore as closest possible resemble the observations *T* ^exp^:

$$q^{*}=\underset{q\in Q}{\mathrm{argmin}}\;\|T^{\exp}-T\left(x\right)\|\text{ s.t. }\left(\begin{array}{c} \ddot{x}\\ \dot{x} \end{array}\right)=\left(\begin{array}{c} F\left(x,\dot{x}\right)⊘m\\ dx/dt \end{array}\right),$$ (3)

where Q is a set of hyperparameters controlling the differential equation’s initial values and constants, i.e., $F\left(x,\dot{x}\right)$ and ***m*** depend on ***q***, and component-wise division is denoted as ⊘. Since $\ddot{x}$ and $\dot{x}$ are variables in an iterative scheme, the notation *d* ***x***/*dt* is used to emphasize the numerical computation of the latter one. The problem itself is non-convex and requires global optimization Döllinger et al. (2002). To our knowledge, no analytical solution of second-order ODE has been found, such that each optimization step enforces numeric reevaluation of the model. We chose $Q\subset R_{>0}^{14}$ as a multiplicative scaling factor set by splitting the six DOFs of the 2 MM, as defined by Gómez et al. (2018), into three longitudinal segments: six reciprocal scaling factors *m* ^−1^ for vertical mass pairs, six anchor spring stiffnesses *k*_*a*_, subglottal pressure *P*_*S*_, and collision force stiffness proportionality *ξ*_*c*_.

### 2.2 Sampling procedure

By definition, q∈Q is a positive vector, and the identity vector q=1 corresponds to the model’s default values (Steinecke and Herzel, 1995; Schwarz et al., 2008). To achieve reasonable distribution symmetry to the default values, scaling within boundaries (*q*_min_, *q*_max_) with less than some arbitrary factor *λ* ≥ 1 should be as likely as scaling by more than *λ* ^−1^. We therefore demand $P\left(q_{i}\leq \lambda \right)=P\left(q_{i}\geq \lambda^{-1}\right)$ for each component ***q***_*i*_, which is satisfied by log-uniform distributions with reciprocally inverse boundaries $\left(q_{\max}=q_{\min}^{-1}\right)$ . Previous optimization methods (Döllinger et al., 2002; Schwarz et al., 2008; Gómez et al., 2018) assumed a hypercubic search space Q , which is, under consideration of the before mentioned symmetry arguments, comparable to log-uniform sampling of the vector component ***q***_*i*_ with probability (Eq. 4)

$$P\left(q_{i}\right)=\frac{1}{q_{i}\left(\log\left(q_{\max}\right)-\log\left(q_{\min}\right)\right)},$$ (4)

using lower and upper boundaries *q*_min_≔5^−1^ and *q*_max_≔5, respectively. The distribution’s median is 1 and therefore corresponds to the models default values, unlike the distribution’s mean, which is $\left(q_{\max}-q_{\min}\right)/\log\left(q_{\max}\cdot q_{\min}^{-1}\right)\approx 1.49$ . To relate the models’ rest positions ***x*** ^*r*^ to the glottis geometry (Eq. 5) by a simple computation available for both synthetic and experimental trajectories, we assumed:

$$x^{r}=\frac{1}{N}\underset{t}{\sum }T\left(x\left(t\right)\right).$$ (5)

The rest positions ***x*** ^*r*^ are not known beforehand but must be distributed like experimental trajectories *T* ^exp^ under the assumption of Eq. 5. The gamma distribution Γ is commonly chosen for modeling skewed data like *T* ^exp^; therefore, we assume the rest positions to be marginally gamma-distributed (Eq. 6):

$$\sigma_{i}\cdot x_{i,j}^{r}∼\Gamma \left(\alpha_{j},\beta_{j}\right)+c_{j}\;\;|\;\;\sigma_{i}≔\left\{\begin{array}{cc} -1,\; & \text{if }i=1\\ 1,\; & \text{else}, \end{array}\right.$$ (6)

where distribution shape *α*_*i*_, scale *β*_*i*_, and shift *c*_*i*_ are estimated experimentally. The auxiliary variable *σ*_*i*_ specifies the side-dependent sign, where we chose left trajectories to be negative.

As the glottis shape is not arbitrary, the rest positions are statistically dependent. In statistical modeling, copulas provide an elegant way to compose dependent univariate marginal distributions into a joint multivariate distribution (Nelsen, 2006). We decided to join the gamma-distributed marginals by a normal copula C (Eq. 7), as samples will share the exact same covariance as the experimental trajectory-wise means:

$$\begin{array}{ll} \text{vec}\left(x^{r}\right) & ∼C\left(x_{1,1}^{r},\ldots ,x_{2,3}^{r};\tilde{\rho }\right)≔\\ & ≔N\left(N^{-1}\left(x_{1,1}^{r}\right),\ldots ,N^{-1}\left(x_{2,3}^{r}\right);\tilde{\rho }\right), \end{array}$$ (7)

where vec(***x*** ^*r*^) denotes vectorization. $N\left(•;\tilde{\rho }\right)$ is the multivariate normal cumulative distribution function (CDF) with zero mean and covariance matrix $\tilde{\rho }≔dI+\left(1-d\right)\rho$ , the inversed standard normal CDF is denoted as $N^{-1}\left(•\right)$ , and *I* is the identity matrix. The correlation matrix $\rho \in R^{6\times 6}$ was estimated for experimental trajectories and is blended toward independence by a control parameter *d* ∈ [0, 1], where *d* = 0.5 was chosen to enlarge sampling diversity.

### 2.3 Convolutional recurrent neural network

By carrying out the sampling procedure, a dataset $D≔\left\{\left(T\left(x^{\left(i\right)}\right),q^{\left(i\right)}\right)\;|\;i=1,\ldots ,|D|\right\}$ is obtained. The trajectories $T\left(x^{\left(i\right)}\right)\in R^{6\times n}$ , which are computed for the 6 MM’s ODE solution ***x*** ^(*i*)^ given the *i*-th sample $q^{\left(i\right)}\in R_{>0}^{14}$ over *n* time steps, serve as synthetic network input data. The associated label ***q*** ^(*i*)^ to be trained against is continuous and strictly positive, such that its inference given *T*(***x*** ^(*i*)^) may be arguably treated as a positive regression problem on time-series data.

Each 6 MM configuration is simulated over *n* = 1000 time steps of 0.25 *ms* physical time, such that each trajectory in the dataset is 250 *ms* in length. The first 75 *ms* are truncated, as we seek to train on the non-transient phase. To avoid potential overfitting, the network inputs are randomly chosen trajectory sub-sequences, of a length of 512 time steps (128 *ms*), which are rerolled in every iteration and degraded by 10% additive Gaussian noise. Furthermore, we found it beneficial to zero-truncate the trajectories as prior input modification, such that left trajectories are signed negative and right trajectories positive.

**FIGURE 3:**
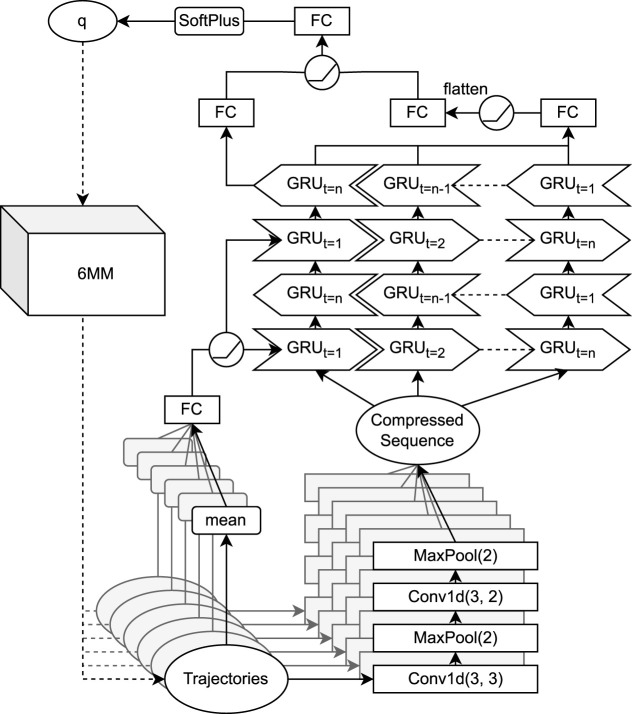
Convolutional recurrent neural network (CRNN) architecture for inverse six-mass model (6 MM) parameter estimation. Convolutional operations in the lower layers are performed trajectory-wise, before the joint sequence is iterated through a bidirectional gated recurrent unit (GRU). Fully connected (FC) layers with rectifier linear unit (ReLU) activation functions are used as top layers.

To solve this time-wise trajectory-based regression problem, we used the following attention-based (Bahdanau et al., 2014) CRNN architecture shown in Figure 3. Each trajectory is compressed by a small sub-CNN for each trajectory, a composite of four layers altering convolutions and max pooling with kernel size 2. Both convolutions have a kernel size of 3, the lower convolution has stride 3 and 10 channels and the upper one has stride 2 and five channels, such that the sub-CNN effectively compresses the six trajectories into a 21 × (5 ⋅ 6) tensor. The particular architecture was loosely motivated by classical CNNs (LeCun et al., 1998) and was adapted to moderate sequence length reduction. Each of the 21 features in the temporally compressed sequence can be attributed to 24 time steps (6 *ms*), respective 30 time steps (7.5 *ms*) without considering kernel overlapping. Here, the temporal resolution was reduced toward the lower wavelength’s magnitude, while the detail is preserved through an increased channel amount. Since the signal is relatively smooth, increasing the stride is generally preferred over that of kernel size.

Next, the compressed data are iterated through a two-layer bidirectional gated recurrent unit (GRU) (Cho et al., 2014) with a hidden size of 256. To incorporate prior knowledge on the importance of trajectory means (cf. Eq. 5), the hidden state is initialized with the trajectory means after being passed through a fully connected (FC) layer and a rectified linear unit (ReLU). For the decision making on the network’s prediction $\hat{q}\in R_{>0}^{14}$ , several FC layers are used to combine sequence-wise RNN output and hidden state. An attention mechanism is supposed to ease decision making on the signals’ long time dependencies. It consists of an FC layer with target length 16 applied to every GRU iteration’s output, such that a 16 × 30 tensor is obtained, which is flattened and again processed by another FC layer with target length 16. Similarly, the top GRU layer’s last hidden state is processed by a single FC layer with target size 16 and is processed to a tensor of size dim(*Q*) by an FC layer after being merged with the sequential attention tensor. Subsequent FC layers are separated through ReLUs. Last, a softmax layer is incorporated to ensure prediction positivity.

Root mean square error (RMSE) loss is commonly used for regression problems, which are defined on an entire real vector space including negative numbers, but in our case, ***q***_*i*_ is a strictly positive multiplicative quantity. The standard regression case is obtained by logarithmization, and for this reason, root mean square logarithmic error (RMSLE) loss (Eq. 8) was used, as a natural adaption to RSME:

$$RMSLE=\sqrt{\frac{1}{N}\overset{N}{\underset{i=1}{\sum }}{\left(\log\left(q_{i}\right)-\log\left({\hat{q}}_{i}\right)\right)}^{2}}$$ (8)

### 2.4 Setup

The 6 MM is implemented in Julia 1.8 (Bezanson et al., 2014), which offers a good performance–convenience trade-off (Churavy et al., 2022). To solve the ODE, the classical fourth-order Runge–Kutta method is used with time step 0.25 *ms* using double precision. The NN is written in PyTorch 1.13 (Paszke et al., 2019) under Python 3.8 (Van Rossum and Drake, 2009), and single precision was used to speed up computations.

Adaptive momentum (Kingma and Ba, 2014) was used to train the network with an exponential decay of learning rate with base 0.9 and initial value 5 ⋅ 10^−3^. Early stopping with a patience of five epochs is used as convergence criteria. A batch size of 512 was used. We computed datasets of varying sizes between 10^4^ (10 k) and 10^6^ (1 M) samples by evaluating the 6 MM (Eq. 2) for randomly sampled 6 MM parameters ***q*** ^(*i*)^ and rest positions ${\left(x^{r}\right)}^{\left(i\right)}$ . A fraction of 10% was split from the obtained 6 MM samples as the validation dataset. As non-transient 6 MM configurations are not accounted in the sampling procedure, we excluded samples with vanishing trajectory oscillation, i.e., trajectories with standard deviation $\text{std}\left(T\left(x^{\left(i\right)}\right)\right)<10^{-3}$ , effectively reducing the training data to 70% of the generated samples.

All computations were executed on an *Intel i9-11900* CPU with 64 GB RAM and an *Nvidia RTX 3070* GPU. The model runs on our machine with a single core speed of approximately 10.5 GFlop/s. Simulating 6 MM-based VF motion for physical 250 ms took 330 *μ*s on our hardware. Randomized sampling of 10 k 6 MM trajectories took about 7.2 s with a two-fold multithreading speed up using eight threads.

Testing data for this work were obtained by the experimental setup of Birk et al. (2017a) and essentially contain HSV recordings (4000 FPS) and subglottal pressure measurements for porcine larynges that have been tested under varying air throughput and different pre-phonatory configurations. Experimental trajectories *T* ^exp^ were obtained from the HSV recordings and were segmented with the software *Glottis Analysis Tools* (Kist et al., 2021). Six larynges, with 288 recordings in total, were selected based on the recording quality by Gómez et al. (2018). Observed subglottal pressure values range from 459 Pa to 1494 Pa and are approximately (Lilliefors test, *p* = 0.946) normal distributed to $P_{S}∼N\left(997\;\text{Pa},227\;\text{Pa}\right)$ , and the sensor’s accuracy was about 35 Pa (Gómez et al., 2019). The pre-phonatory configurations include symmetric (Birk et al., 2017b) and asymmetric (Semmler et al., 2021) arytenoid torques (5–25 m Nm). Furthermore, the rest positions were affected by differing posterior gaps used in the setup: in 140 recordings, a 1 mm metal shim was inserted between arytenoid cartilages; in 95 recordings, a 2 mm shim was used, and the remaining 53 recordings were unmodified. No experimental estimation of tissue characteristics like mass and stiffness was performed in the setup.

Calibration to metric units was done implicitly by scaling the recorded anterior–posterior distance to the VF elongation, which was set to 4 ⋅ *ℓ*_*l*_ = 2 cm to match the porcine testing data. The fixed ends of the longitudinal anchor springs were (laterally) located at ± 0.05 mm in the posterior position and were 0 in the anterior position. For the remaining model, parameters were set to *k*_*v*_ = 1 mm, *r*_*a*_ = 0.0002/3 Nsm^−1^, and *ℓ*_*v*_ = 2 mm. Non-zero initial deflections ***x*** (*t*) = ±1 mm were assumed for the lower masses.

### 2.5 Glottal geometry fitting

**FIGURE 4:**
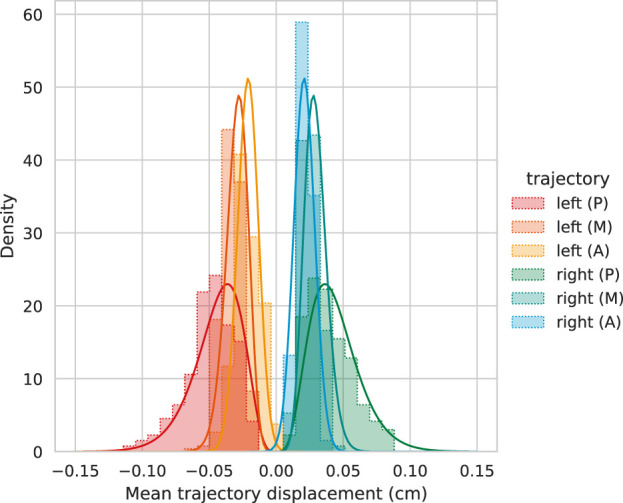
Statistical analysis for time-wise averaged experimental trajectories (A = anterior, M = medial, and P = posterior). The fitted gamma distributions Γ(*α*, *β*)+ *c* match the histograms sufficiently.

For the sampling procedure (cf. Section 2.2), gamma-distributed rest position marginals were used. As can be seen in Figure 4, the averaged experimental trajectory distribution shows skewness of different magnitude: moderate skewness was observed in the posterior direction, 0.71; shrinking to slight medial skewness, 0.30; and vanishingly small skewness −0.07 in the anterior direction. To suppress undesired lateral asymmetry observations by the experimental setup, left and right trajectories are not distinguished, i.e., computations were performed on (2 ⋅ 288) × 3 positively oriented trajectories. In every case, gamma distribution Γ(*α*, *β*) + *c* fitting was acceptable for our purpose, and exact fitting parameters are found in Table 1. The fitted distribution’s skewness is most prominent in posterior positions and decreases for larger shape parameters *α* toward the anterior direction, such that the distribution’s Gaussianity is increased. Larger skewness in the posterior direction should be partially attributed to the different pre-phonatory configurations in the experimental setup, where a posterior gap was induced by a metal shim.

**TABLE 1:** Gamma distribution Γ(*α*, *β*)+ *c* fitting parameters with shape *α*, rate *β*, and shift *c* for experimental trajectories. The fitted distributions (A = anterior, M = medial, and P = posterior) are used to sample the model’s resting positions for the synthetic training data.

	*α*	*β*	*c*
P	5.59 ⋅ 10^0^	7.96 ⋅ 10^−3^	−1.66 ⋅ 10^−4^
M	2.38 ⋅ 10^1^	1.70 ⋅ 10^−3^	−1.11 ⋅ 10^−2^
A	1.45 ⋅ 10^3^	2.05 ⋅ 10^−4^	−2.76 ⋅ 10^−1^

**FIGURE 5:**
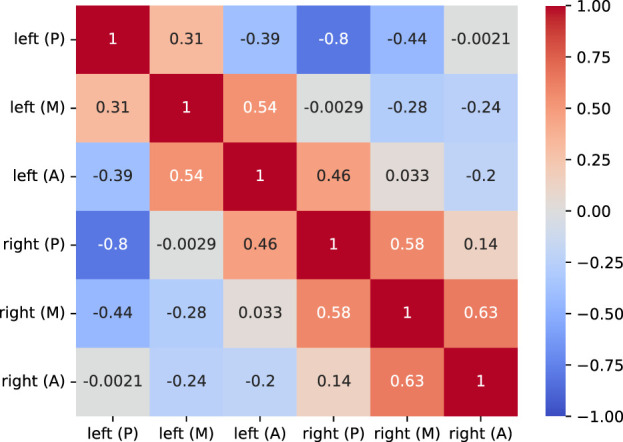
Correlation matrix *ρ* of the time-wise averaged experimental trajectories. Off-diagonal non-zero elements imply statistical dependency in the data, and negative values indicate anti-correlation.

In addition to skewness, the experimental (signed) trajectories show statistical dependencies, visualized as the correlation matrix in Figure 5. The medial trajectory mean correlation between 0.31 and 0.63 to the anterior and posterior direction was observed for both VF sides, while opposite trajectories are anti-correlated with values ranging from −0.2 for the anterior to −0.8 for the posterior direction as expected. Expecting a side-wise block form, the correlation matrix shows an anomaly involving the left anterior. The left anterior mean correlates to the opposite posterior with 0.46 but anti-correlates to the left posterior with −0.39. In summary, many rest positions moderately to strongly correlate and anti-correlate to the opposing side in an expected way, and the left anterior is off.

## 3 Results

In the following section, the results on the network’s performance against synthetic and experimental data are presented. Furthermore, the network’s predictive capabilities on different parameters are analyzed.

### 3.1 Parameter learning

**FIGURE 6:**
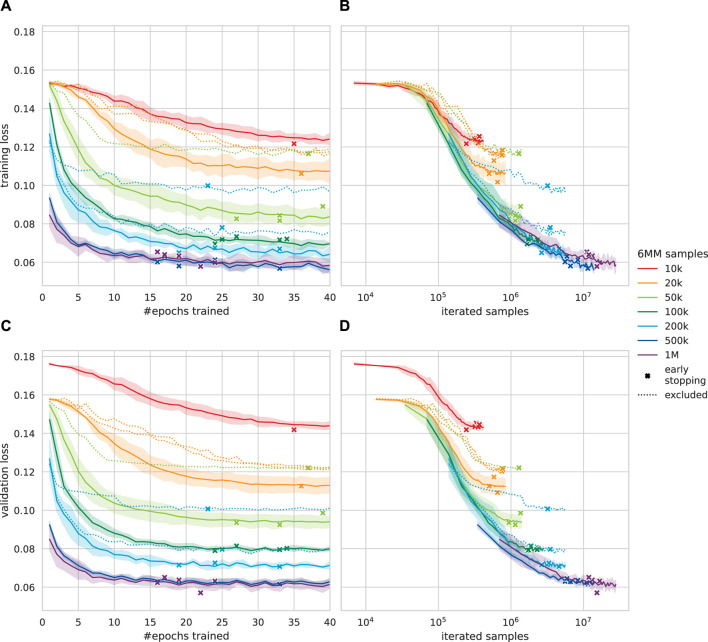
Root mean squared logarithmic error (RMSLE)-based loss curves (less is better) for the presented convolutional recurrent neural network (CRNN), trained on different amounts of six-mass model (6 MM) samples. For each training dataset, five CRNNs were trained. Iterated samples are computed as the amount of training data times epochs trained. Standard deviation is indicated by the translucent area. **(A)** Epoch-wise training loss. **(B)** Computational effort against training loss. **(C)** Epoch-wise validation loss. **(D)** Computational effort against validation loss.

To judge the CRNN’s ability to learn the model’s VF kinematics, we performed five-fold training of the architecture on 6 MM parameter reproduction for each of the 6 MM datasets, excluding about 23% non-oscillating samples. In Figure 6, RMSLE-based validation loss curves for different dataset sizes are shown. Tendential, smaller datasets required more than 40 epochs to converge (early stopping with a patience of five epochs, cf. Section 2.4), whereas larger datasets converged in around 20 epochs, and the exact values can be found in Table 2. An increased training data amount leads to epoch-wise faster validation loss reduction as the network iterates more samples per epoch. By comparing validation loss against the total number of iterated samples, it can be seen that less samples are not sufficient to reduce the validation loss to a magnitude comparable with that of larger training sets. The training loss was about 2%–15% lower than the validation loss for 1 M and 10 k samples, respectively. For all sampling sizes, each run whose validation loss was worse by more than 10% than the best run was excluded. A total of six subperformant candidate networks, which converged to significantly worse validation loss values, were excluded from further analysis. The affected runs were not representative, i.e., the convergence problems did not affect all tested sampling sizes equally, and including networks with convergence problems was found obstructive for clear trends in the following evaluation.

**TABLE 2:** Mean absolute error (MAE) for each predicted six-mass model (6 MM) parameter on synthetic validation data. Validation loss-wise subperformant networks were excluded. Optimal statistical guessing results in a 0.994 MAE.

6 MM samples			10 k	20 k	50 k	100 k	200 k	500 k	1 M
Training data			6920	13,872	34,773	69,633	139,202	348,016	695,747
Epochs			45.6 ± 7.0	40.7 ± 5.5	32.0 ± 4.9	27.6 ± 4.6	24.3 ± 7.1	20.8 ± 7.0	18.6 ± 3.4
Validation loss			0.144 ± 0.001	0.113 ± 0.004	0.094 ± 0.003	0.080 ± 0.001	0.072 ± 0.001	0.063 ± 0.001	**0.062 ± 0.003**
*m* ^−1^	Left	P	0.840 ± 0.036	0.743 ± 0.052	0.611 ± 0.024	0.452 ± 0.011	0.377 ± 0.034	0.299 ± 0.017	**0.276 ± 0.021**
		M	0.883 ± 0.022	0.847 ± 0.084	0.612 ± 0.035	0.535 ± 0.024	0.462 ± 0.017	0.397 ± 0.018	**0.343 ± 0.012**
		A	0.985 ± 0.026	0.753 ± 0.076	0.644 ± 0.028	0.517 ± 0.013	0.443 ± 0.021	0.366 ± 0.018	**0.335 ± 0.024**
	Right	P	0.916 ± 0.047	0.815 ± 0.026	0.669 ± 0.027	0.537 ± 0.014	0.487 ± 0.016	0.399 ± 0.023	**0.372 ± 0.025**
		M	0.862 ± 0.046	0.779 ± 0.053	0.610 ± 0.021	0.525 ± 0.018	0.463 ± 0.018	0.395 ± 0.018	**0.342 ± 0.015**
		A	0.895 ± 0.039	0.783 ± 0.160	0.616 ± 0.014	0.516 ± 0.010	0.435 ± 0.019	0.369 ± 0.020	**0.334 ± 0.018**
*k*	Left	P	0.683 ± 0.074	0.539 ± 0.059	0.478 ± 0.013	0.364 ± 0.015	0.311 ± 0.030	0.246 ± 0.013	**0.234 ± 0.017**
		M	0.682 ± 0.051	0.557 ± 0.114	0.483 ± 0.017	0.433 ± 0.010	0.381 ± 0.008	0.324 ± 0.013	**0.282 ± 0.006**
		A	0.709 ± 0.023	0.557 ± 0.069	0.476 ± 0.023	0.406 ± 0.010	0.344 ± 0.017	0.299 ± 0.015	**0.271 ± 0.018**
	Right	P	0.761 ± 0.119	0.673 ± 0.058	0.523 ± 0.034	0.421 ± 0.019	0.368 ± 0.016	0.313 ± 0.024	**0.292 ± 0.020**
		M	0.760 ± 0.132	0.640 ± 0.088	0.508 ± 0.027	0.419 ± 0.016	0.373 ± 0.014	0.324 ± 0.014	**0.287 ± 0.010**
		A	0.605 ± 0.013	0.537 ± 0.081	0.484 ± 0.021	0.422 ± 0.004	0.350 ± 0.016	0.302 ± 0.015	**0.276 ± 0.018**
*P*_*S*_			0.322 ± 0.013	0.263 ± 0.033	0.185 ± 0.009	0.148 ± 0.010	0.120 ± 0.003	0.093 ± 0.005	**0.072 ± 0.004**
*ξ*_*c*_			1.019 ± 0.012	0.932 ± 0.068	0.995 ± 0.009	0.953 ± 0.008	0.937 ± 0.058	**0.834 ± 0.007**	0.951 ± 0.098

Best values are indicated bold.

Even though RMSLE validation loss equally weights each component $q_{j}^{\left(i\right)}$ of the label vector ***q*** ^(*i*)^ and all components are sampled independently and identically distributed, we observed significant differences in the prediction’s MAE $\left|q_{j}^{\left(i\right)}-{\hat{q}}_{j}^{\left(i\right)}\right|$ shown in Table 2. Each quantity besides the collision force proportionality *ξ*_*c*_ was continuously improved by increasing the amount of synthetic data. The subglottal pressure *P*_*S*_ was predicted most accurately, and stiffness *k*_*a*_ was learned slightly better than reciprocal mass *m* ^−1^ in most cases, while *ξ*_*c*_ was the least learnable parameter in every scenario. An assessment on optimal statistical guessing can be found in Section 4.3. For each parameter, a unitless value of 1.0 corresponds to the model’s default value in real units. The corresponding metric MAEs and sampling interval boundaries for 1 M samples are given in Table 3, including general model specifications. Changing the hypercube size of Q did not improve the results; for comparison purposes, equivalent boundaries were preferred.

**TABLE 3:** Model specifications in metric units and mean absolute error (MAE) for predicted six-mass model (6 MM) parameters on synthetic validation data. The averaged MAEs were obtained by evaluating the predicted 6 MMs of all networks trained with 1 M samples. Physical specifications for mass and stiffness vary vertically (B = bottom; T = top). The average stiffness of two adjacent anchor springs is denoted as $2{\bar{k}}_{a}$ .

		***q***_*i*_	*m*	*k*_*a*_	*P*_*S*_	*ξ*_*c*_	*k*_*c*_	*ξ*_*l*_	*k*_*l*_	*k*_*v*_	*r*_*a*_	*ℓ*_*l*_	*ℓ*_*v*_
			[*g*]	[*Nm* ^−1^]	[*Pa*]		[*Nm* ^−1^]		[*Nm* ^−1^]	[*Nm* ^−1^]	[*Nsm* ^−1^]	[*cm*]	[*cm*]
Default	B	1	0.125/3	80/3	800	1	80*ξ*_*c*_	0.2	$2{\bar{k}}_{a}\xi_{l}$	0.1	0.0002/3	0.5	0.2
	T		0.025/3	8/3			8*ξ*_*c*_						
min	B	0.2	0.00833	5.33	160	0.2							
	T		0.00166	0.533									
max	B	5	0.208	133	4,000	5.0							
	T		0.0416	13.3									
Mean	B	1.49	0.0621	39.8	1200	1.49							
	T		0.0124	3.98									
MAE	B	0.333	0.0121	7.67	57.5	0.951	79.5		2.02				
	T		0.00242	0.767			7.95		0.202				

**FIGURE 7:**
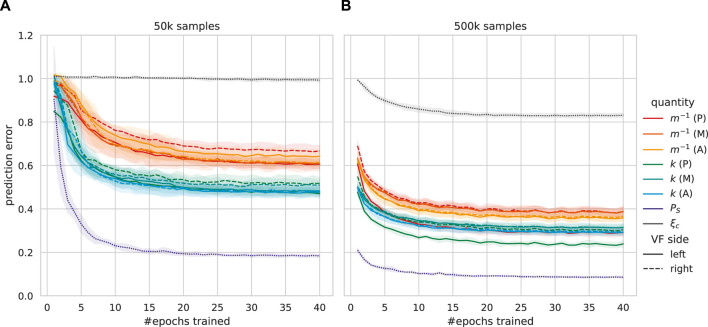
Validation data-based learnability of each six-mass model (6 MM) parameter ***q***_*i*_ as mean absolute error (MAE, lower is better) over the training progress of five networks. Each error is stochastically decreased by training more epochs, and the translucent area indicates standard deviation. Each vocal fold (VF) is divided longitudinally (A = anterior, M = medial, and P = posterior). **(A)** Training with 50 k 6 MM samples, excluding one validation loss-wise subperformant network. **(B)** Training with 500 k 6 MM samples.

The learning progress of each quantity ***q***_*i*_ is representatively visualized for 50 k and 500 k samples in Figure 7. Subglottal pressure *P*_*S*_ already shows substantial improvements after the first few epochs, even for the comparably small 50 k sample dataset, the MAE was reduced to roughly 0.4 within two epochs and again halved within 40 epochs, while stiffness and reciprocal masses spread above 0.47. The collision force proportionality *ξ*_*c*_ did not show convergence at all for 50 k samples, and each other quantity was learned within few epochs using 500 k to 1 M samples, with up to 50% improvement over 50 k samples.

### 3.2 Experimental data testing

Both the synthetic training and validation data share the same characteristics, as they were generated by the same procedure. On the contrary, our testing data stem from the *ex vivo* porcine experiments described in Section 2.4 and therefore characteristically differ from the synthetic data. By this means, the networks’ predictive capabilities on synthetic data cannot directly be transferred to experimental data, such that better accuracy on synthetic data does not necessarily imply an improvement in experimental data.

Validation loss is a measure for the network’s predictive performance on validation data; however, there is no equivalent metric on experimental data. Instead, we judge the prediction by the MAE of three observable measures: the subglottal pressure *P*_*S*_, which was directly measured by a pressure sensor below the larynx, as well as fundamental frequency *f*_0_ and amplitude for each HSV-recorded trajectory. For simplicity, *f*_0_ > 50 Hz is estimated as the maximum of a reciprocally scaled (overtone suppression) Fourier spectrum, and the amplitude is defined as max(*T*_*i*,*j*_(***x***)). If a sole amplitude or frequency is stated, the six trajectories’ estimates were averaged.

Results on the experimental data, using the candidate networks from Section 3.1, are shown in Table 4. On average, 10 k 6 MM samples performed the worst for all metrics, and 50 k samples were slightly best for *f*_0_ and amplitude estimation, but there is no clear preference. The most accurate *P*_*S*_ predictions were achieved by 100 k to 1 M samples. Mean absolute percentage error (MAPE) comparison shows that *f*_0_ was generally estimated more accurately than *P*_*S*_, while the amplitude was the worst estimate for 100 *k* and above samples. The subglottal pressure sensor accuracy was 35 Pa, the measurement uncertainty is negligible for *f*_0_, and the amplitude due to segmentation is very less, such that each reported error is significantly larger than the measurement uncertainty.

**TABLE 4:** Mean absolute error (MAE) and mean absolute percentage error (MAPE) for averaged predictions on experimental data. Validation loss-wise subperformant networks were excluded.

	MAE			MAPE		
6 MM samples	*P*_*S*_ [Pa]	*f*_0_ [Hz]	Amplitude [mm]	*P*_*S*_	*f*_0_	Amplitude
10 k	293 ± 60	27.3 ± 7.8	0.156 ± 0.006	30.4% ± 6.6%	19.3% ± 4.2%	23.3% ± 0.9%
20 k	238 ± 66	15.8 ± 2.7	0.145 ± 0.005	25.2% ± 7.3%	11.5% ± 1.7%	22.1% ± 0.6%
50 k	188 ± 21	**14.8 ± 2.0**	**0.132 ± 0.004**	19.2% ± 2.2%	**10.1% ± 1.2%**	**20.5% ± 0.6%**
100 k	158 ± 13	17.1 ± 1.7	0.139 ± 0.004	16.6% ± 1.4%	11.1% ± 1.0%	22.0% ± 0.7%
200 k	**150 ± 8**	17.5 ± 3.0	0.141 ± 0.003	**15.7% ± 0.7%**	11.6% ± 1.8%	22.7% ± 0.7%
500 k	155 ± 30	15.4 ± 1.0	0.147 ± 0.003	16.3% ± 3.0%	**10.1% ± 0.4%**	23.8% ± 0.5%
1 M	164 ± 13	17.2 ± 1.4	0.147 ± 0.006	16.7% ± 1.2%	11.1% ± 1.0%	23.7% ± 0.9%

Best values are indicated bold.

**FIGURE 8:**
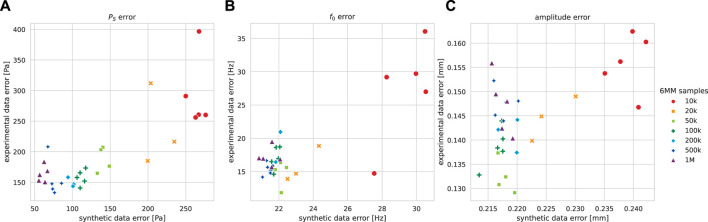
Prediction mean absolute error (MAE) on experimental and synthetic data (lower is better) for five independently trained networks for each dataset size, excluding validation loss-wise subperformant runs. **(A)** Subglottal pressure *P*_*S*_. **(B)** Fundamental frequency *f*_0_. **(C)** Amplitude.

The transferability of synthetic data results to the experimental data is visualized for observable prediction quality estimates in Figure 8, where each network’s prediction accuracy on synthetic trajectories is compared to the network’s corresponding accuracy on experimental trajectories. High characteristic similarity of synthetic and experimental trajectories is expected to result in approximately equivalent performance on both kinds. Training with an increased 6 MM sample amount generally reduced the synthetic errors for each quantity. Increased *P*_*S*_ accuracy on synthetic data tendentially improved the accuracy on experimental data, except for networks trained with 1 M samples and one 500 k run. The largest discrepancy was observed for 1 M samples, where the experimental data MAE was about threefold the synthetic data MAE. For the fundamental frequency and amplitude predictions of networks trained with up to 50 k samples, synthetic data improvements were roughly transferable to those of the experimental data. In both cases, training with more 100 k samples worsened the experimental accuracy; however, the MAE was better than on synthetic data. While higher synthetic MAEs are irritating in the first place, more trajectory varieties must be accounted for the synthetic data due to the sampling procedure’s broad mass–stiffness combination variety. Each observed trend also appears if the MAPE is considered instead of MAE.

**FIGURE 9:**
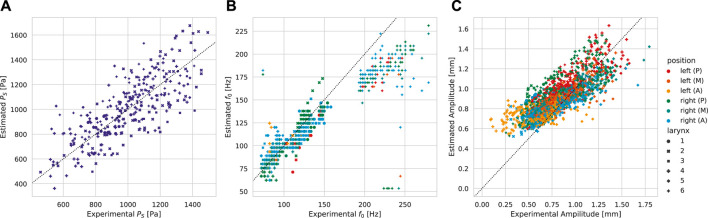
Prediction vs. ground truth correlation analysis for subglottal pressure, fundamental frequency, and amplitude given the subglottal pressure-wise best network. Trajectories (A = anterior, M = medial, and P = posterior) were obtained by evaluating the 6 MM with the predicted parameters. The dashed 1:1 line indicates perfect matching. **(A)** Subglottal pressure *P*_*S*_. **(B)** Trajectory fundamental frequencies *f*_0_. **(C)** Trajectory amplitudes.

**FIGURE 10:**
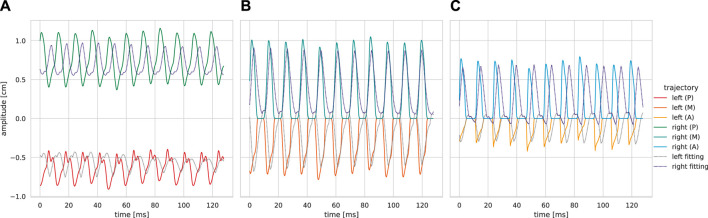
Exemplary trajectory fittings for a single recording. The fitted trajectories have been obtained by simulating the 6 MM with the estimated model parameters of subglottal pressure-wise best candidate network. **(A)** Posterior. **(B)** Medial. **(C)** Anterior.

On the experimental ground truth, the best subglottal pressure predictions (*P*_*S*_ = 133 Pa) were achieved by a candidate NN that was trained for 24 epochs using 500 k 6 MM samples. The predictions are scattered against the ground truth in Figure 9, and the network accuracy on experimental data can be found in Table 5. A 76.6% correlation between prediction and ground truth was achieved for the subglottal pressure estimate. Low fundamental frequency predictions show a good correlation, while high fundamental frequencies (measured for the 6th larynx) were underestimated for medial and posterior positions. For about 10 samples, the frequencies were mispredicted completely. Amplitude predictions concentrate along the 1:1 matching line with moderate scattering, but small amplitudes were overestimated. Estimations for different experiments with the same larynx were adjacent frequently, which, for example, can be seen in the left anterior predictions for larynx 4. A visual impression on exemplary trajectory fitting using the best candidate network is given in Figure 10. The medial trajectories were estimated most closely with good *f*_0_ accuracy. Characteristically, anterior trajectories were fitted at a desirable level, and similarly for the posterior trajectories, the phase does not match. Experimental and fitted posterior trajectories both show incomplete glottis closure of the same level.

**TABLE 5:** Best-case mean absolute error (MAE) and mean absolute percentage error (MAPE) for the predictions on experimental data using a network trained with 500 k samples for 24 epochs.

		MAE			MAPE		
		*P*_*S*_ [Pa]	*f*_0_ [Hz]	Amplitude [mm]	*P*_*S*_	*f*_0_	Amplitude
Left	P		14.9 ± 16.4	0.162 ± 0.091		9.1% ± 7.2%	19.8% ± 13.6%
	M		14.6 ± 19.2	0.098 ± 0.082		8.6% ± 7.7%	12.4% ± 11.6%
	A		17.3 ± 19.7	0.156 ± 0.143		11.4% ± 9.4%	40.5% ± 73.4%
Right	P		16.4 ± 25.0	0.201 ± 0.109		9.9% ± 13.0%	28.9% ± 21.8%
	M		14.5 ± 19.5	0.127 ± 0.087		8.9% ± 11.1%	17.6% ± 16.2%
	A		17.4 ± 20.2	0.120 ± 0.104		11.7% ± 11.9%	18.9% ± 21.2%
Average		133 ± 97	15.9 ± 20.2	0.144 ± 0.110	13.9% ± 11.3%	9.9% ± 10.4%	23.0% ± 35.1%

## 4 Discussion

In this section, the CRNN’s accuracy on synthetic and experimental data is compared to statistic guessing and related work. In addition to prediction results, the 6 MM’s computational performance and rest-position sampling are discussed. Lastly, a brief summary on modeling limitations and an outlook on future work are given.

### 4.1 Execution speed

Schwarz et al. (2008) reported a computational real-time ratio of almost 1:1, i.e., simulating their C# 6 MM for 1 s physical time with 0.25 ms time step takes 1s on an *Intel Pentium 4* based setup. By implementing the simplified 6 MM in a fast executing programming language, a more than 750-fold single-core speedup was realized on a modern *Intel i9-11900* processor. The Julia 6 MM also surpassed the 100:1 real-time ratio, reported for the less complex 2 MM by Gómez et al. (2019) using an *Intel i5-4590* processor. With consideration of different processor single-core capabilities, the presented Julia 6 MM remains the fastest. Easing 6 MM computing time to fractions of milliseconds, the computational necessity to use less physiological models for speed becomes obsolete. For efficient trajectory-based training, we found CRNN-based architectures beneficial over plain RNNs, as sequences are compressed by computationally superior convolution operations.

### 4.2 Rest-position geometry

During this work, we found constant spring resting positions ***x*** ^*r*^ in synthetic training data generation to be vastly obstructive for realistic model predictions. When using trajectory-unspecific ***x*** ^*r*^, we concluded that the network learned to exploit the damping relation $\frac{r}{2\sqrt{mk}}$ (harmonic oscillator) to compensate the incorrect glottis geometry. By incorporating randomized rest positions into our sampling procedure, we enabled the network to automatically adapt to different values. To encounter dependencies between the rest positions, copulas were chosen as the theoretical construct to join marginals. However, the benefit over training with assumed independence was not significant in our experiments. A possible explanation is that the CRNN generalizes equivalently well in both cases.

### 4.3 Neural network-based predictions

For synthetic validation data, the model’s learnability by a NN can be judged by comparison against the optimal statistical guess (Eqs 9–12) as trivial lower bound prediction. For a log-uniform distributed random variable *q* on positive interval (*a*, *b*), the MAE of a statistical guess *u* ∈ (*a*, *b*) is

$$E\left[\left|q-u\right|\right]=\int_{a}^{b}|q-u|\frac{1}{q\log\left(\frac{b}{a}\right)}dq$$ (9)

$$=\int_{u}^{b}\frac{q-u}{q\log\left(\frac{b}{a}\right)}dq+\int_{u}^{a}\frac{q-u}{q\log\left(\frac{b}{a}\right)}dq,$$ (10)

$$=\left({\left[q-u\log\left(q\right)\right]}_{u}^{b}+{\left[q-u\log\left(q\right)\right]}_{u}^{a}\right)\frac{1}{\log\left(\frac{b}{a}\right)},$$ (11)

$$=\left(b+a+u\left(\log\left(\frac{u^{2}}{ab}\right)-2\right)\right)\frac{1}{\log\left(\frac{b}{a}\right)}.$$ (12)

The optimal statistical guess is the distribution median (Lee, 1995), i.e., $u^{*}=\sqrt{q_{\min}q_{\max}}=1$ with $E\left[\left|q-u^{*}\right|\right]\approx 0.994$ . Except for the collision force proportionality *ξ*_*c*_, the network’s predictions were substantial improvements in each case. Subglottal pressure was the most learnable variable, which is in line with the observations on synthetic data by Zhang (2020). Using a feedforward neural network (FFNN) featuring three hidden layers and 150 neurons each, trained on biomechanical body cover VF model features with *P*_*S*_ interval 50–2400 Pa, they achieved 0.206 MAE (137.3 Pa) on synthetic validation data. Despite differences in the approach, the value is comparable to our unitless 0.185 MAE (148 Pa) for 50 k 6 MM samples and is surpassed for 1M samples with 57.5 Pa.

As expected, by retraining the CRNN architecture with varying dataset sizes between 10 k and 1 M, we found that the predictions on the synthetic data were continuously improved by increasing the training data amount (cf. Table 2). Testing multiple randomly trained candidate NNs on experimental data was necessary, as small validation loss values did not guarantee *P*_*S*_ error reduction. Likely, the lack of improvements on experimental data in Table 4, in contrast to those in Table 2, is due to model–reality discrepancy, such that further prediction improvements on the synthetic model become meaningless for experimental data beyond a certain training effort. In some cases, the overconfidence in the synthetic model by training too many epochs even lead to worse subglottal pressure prediction on the experimental data, as can be seen in Figure 8. In the much different setup of Ibarra et al. (2021), who used a neck-surface vibrometer to predict subglottal pressure for data from a human *in vivo* study, similar model–reality divergence was reported. Their best predictions on experimental data (191 Pa MAE) were realized by a minimal FFNN with two hidden layers and solely four neurons each, while larger networks, which more than halved the MAE on synthetic data, performed significantly worse on real data. Caution is necessary for a direct comparison to the presented approach, as the 6 MM and related models neglect coupling effects of the vocal tract and clinical limitations like varying camera angles and video calibrations.

For the experimental data, there was no single best candidate network which performed optimally on metrics (*P*_*S*_, *f*_0_, and amplitude). One of the best-case NNs that we found achieved an MAE of 133 Pa on experimental data. Considering an average 74.4 Pa MAE using 500 k samples for validation data again highlights limitations in the 6 MM’s realism. In the 288 *ex vivo* porcine recording dataset, our 133 Pa MAE is a substantial improvement to 2 MM pressure estimation error using an RNN-based approach by Gómez et al. (2019) with 192 Pa MAE and to 172 Pa MAE for their preceding optimization approach (Gómez et al., 2018). For a single excised human larynx, Zhang (2020) reported slightly better 115 Pa; however, for meaningful comparison, re-evaluation on the same dataset would be needed.

For their 2 MM optimization approach, Gómez et al. (2018) reported an amplitude MAE of 0.08 mm and 0.02 Hz MAE for fundamental frequency. The amplitude error is slightly better than the 0.09 mm−0.13 mm for medial trajectories found by our approach. While the optimization’s objective primarily targets the fundamental frequency, the NN only indirectly learns 6 MM oscillation properties, such that frequency and phase prediction are significantly better using optimization.

### 4.4 Modeling limitations

Schwarz et al. (2008) defined the 6 MM’s free spring elongation *ℓ*_•_ as the distance between resting positions, while Steinecke and Herzel (1995) indirectly assumed *ℓ*_•_ = 0 for their 2 MM. With the 6 MM definition, the lateral coupling force component between masses with unidirectional movement freedom and distance *ℓ*_•_ becomes ${\left[F^{•}\right]}_{x}=\left(1-\frac{\ell_{•}}{\sqrt{x^{2}+\ell_{•}^{2}}}\right)k_{•}x$ , compared to ${\left[F^{•}\right]}_{x}=k_{•}x$ for *ℓ*_•_ = 0 in the 2 MM. Among various formulations tried, our approach worked best by applying the entire coupling force $\|F^{•}\|=k_{•}\left(\sqrt{x^{2}+\ell_{•}^{2}}-\ell_{•}\right)$ laterally. Whether this should be understood as force-wise change of direction or the formula better resembles the tissue with *ℓ*_•_ = 0 could not be answered within the scope of this work.

As mentioned in the introduction, the 6 MM and related models do not account for acoustic coupling with the vocal tract. In addition, reality aspects like non-orthogonal camera angles, non-steady phonation, calibration of glottis length, and segmentation problems due to insufficient illumination must be taken into account before a clinical application of the method becomes possible. Even though the results on the tested porcine larynges were desirable, the CRNN is sensitive to outliers like most neural networks. Hence, each aspect could add untrained particularities to the trajectories, which could in the worst case significantly worsen predictions. The stability of the method can likely be increased by averaging multiple runs or by directly incorporating uncertainty with Bayesian deep learning.

### 4.5 Conclusion and future work

By employing a state-of-the-art CRNN architecture, we were able to substantially improve the subglottal pressure prediction results of Gómez et al. (2019). Through further prediction of mass and stiffness, we were able to indirectly predict trajectories, which we judged by frequency and amplitude, through 6 MM re-evaluation. Methodically, this brings NN-based predictions closer to optimization, which is necessary for future combined approaches and helpful for qualitative judgment against optimization-based approaches. By stating detailed results on differing parameter learnability, and the training effort-based prediction error given experimental data, we contributed VF-specific knowledge on stochastic training effects and model–reality discrepancy.

## Data Availability

Publicly available datasets were analyzed in this study. The Neural Networks will be found here: Donhauser et al. (2024), https://doi.org/10.5281/zenodo.10640764. The testing data will be found here: Birk et al. (2024), https://doi.org/10.5281/zenodo.10640031.

## References

[B1] Bahdanau D. Cho K. Bengio Y. (2014). Neural machine translation by jointly learning to align and translate. *CoRR* abs/1409.0473.

[B2] Bengio Y. Simard P. Y. Frasconi P. (1994). Learning long-term dependencies with gradient descent is difficult. IEEE Trans. neural Netw. 5 (2), 157–166. 10.1109/72.279181 18267787

[B3] Bezanson J. Edelman A. Karpinski S. Shah V. B. (2014). Julia: a fresh approach to numerical computing. SIAM Rev. 59, 65–98. 10.1137/141000671

[B63] Birk V. Semmler M. Donhauser J. Gómez P. Schützenberger A. Döllinger M. (2024). Subglottal pressure experiments with ex-vivo porcine larynges [Data set]. Zenodo. 10.5281/zenodo.10640031

[B4] Birk V. Döllinger M. Sutor A. Berry D. A. Gedeon D. Traxdorf M. (2017a). Automated setup for *ex vivo* larynx experiments. J. Acoust. Soc. Am. 141 (3), 1349. 10.1121/1.4976085 28372097 PMC6909984

[B5] Birk V. Kniesburges S. Semmler M. Berry D. A. Bohr C. Döllinger M. (2017b). Influence of glottal closure on the phonatory process in *ex vivo* porcine larynges. J. Acoust. Soc. Am. 142 (4), 2197. 10.1121/1.5007952 29092569 PMC6909995

[B6] Birkholz P. (2011). “A survey of self-oscillating lumped-element models of the vocal folds,” in Studientexte zur Sprachkommunikation: elektronische Sprachsignalverarbeitung 2011. Editors Kröger B. J. Birkholz P. (TUDpress, Dresden: Technische Universität Dresden), 47–58.

[B7] Björklund S. Sundberg J. (2016). Relationship between subglottal pressure and sound pressure level in untrained voices. J. voice official J. Voice Found. 30 (1), 15–20. 10.1016/j.jvoice.2015.03.006 25913752

[B8] Brauwers G. Frasincar F. (2022). A general survey on attention mechanisms in deep learning. IEEE Trans. Knowl. Data Eng. 35, 3279–3298. 10.1109/tkde.2021.3126456

[B9] Cho K. van Merrienboer B. Gülçehre Ç. Bahdanau D. Bougares F. Schwenk H. (2014). “Learning phrase representations using rnn encoder–decoder for statistical machine translation,” in Conference on Empirical Methods in Natural Language Processing.

[B10] Churavy V. Godoy W. F. Bauer C. Ranocha H. Schlottke-Lakemper M. Rass L. (2022). Bridging hpc communities through the julia programming language. ArXiv abs/2211.02740.

[B11] Cielo C. A. Schwarz K. Finger L. S. de Moraes Lima J. P. Christmann M. K. (2019). Glottal closure in women with no voice complaints or laryngeal disorders. Int. Archives Otorhinolaryngology 23, e384–e388. 10.1055/s-0038-1676108 PMC6805197 31649756

[B12] Döllinger M. Berry D. A. Kniesburges S. (2016). Dynamic vocal fold parameters with changing adduction in ex-vivo hemilarynx experiments. J. Acoust. Soc. Am. 139, 2372–2385. 10.1121/1.4947044 27250133 PMC4859834

[B13] Döllinger M. Hoppe H. U. Hettlich F. Lohscheller J. Schuberth S. Eysholdt U. (2002). Vibration parameter extraction from endoscopic image series of the vocal folds. IEEE Trans. Biomed. Eng. 49, 773–781. 10.1109/TBME.2002.800755 12148815

[B14] Döllinger M. Tayama N. Berry D. A. (2005). Empirical eigenfunctions and medial surface dynamics of a human vocal fold. Methods Inf. Med. 44, 384–391. 10.1055/s-0038-1633981 16113761

[B15] Döllinger M. M. Berry D. A. Huttner B. Bohr C. (2011). Assessment of local vocal fold deformation characteristics in an *in vitro* static tensile test. J. Acoust. Soc. Am. 130 (2), 977–985. 10.1121/1.3605671 21877810 PMC3190661

[B62] Donhauser J. Tur B. Döllinger M.(2024). Neural network based estimation of biomechanical vocal fold parameters. Zenodo. 10.5281/zenodo.10640764 PMC10916882 38449783

[B16] Erath B. D. Zañartu M. Stewart K. C. Plesniak M. W. Sommer D. E. Peterson S. D. (2013). A review of lumped-element models of voiced speech. Speech Commun. 55, 667–690. 10.1016/j.specom.2013.02.002

[B17] Falk S. Kniesburges S. Schoder S. Jakubaß B. Maurerlehner P. Echternach M. (2021). 3d-fv-fe aeroacoustic larynx model for investigation of functional based voice disorders. Front. Physiology 12, 616985. 10.3389/fphys.2021.616985 PMC7982522 33762964

[B18] Fawaz H. I. Forestier G. Weber J. Idoumghar L. Muller P.-A. (2018). Deep learning for time series classification: a review. Data Min. Knowl. Discov. 33, 917–963. 10.1007/s10618-019-00619-1

[B19] Foumani S. N. M. Miller L. Tan C. W. Webb G. I. Forestier G. Salehi M. (2023). Deep learning for time series classification and extrinsic regression: a current survey. ArXiv abs/2302.02515.

[B20] Fraile R. Kob M. Godino-Llorente J. I. Sáenz-Lechón N. Osma-Ruiz V. J. Gutiérrez-Arriola J. M. (2012). Physical simulation of laryngeal disorders using a multiple-mass vocal fold model. Biomed. Signal Process. Control. 7, 65–78. 10.1016/j.bspc.2011.04.002

[B21] Fukahori M. ichi Chitose S. Sato K. Sueyoshi S. Kurita T. Umeno H. (2016). Regeneration of vocal fold mucosa using tissue-engineered structures with oral mucosal cells. PLoS ONE 11, 0146151. 10.1371/journal.pone.0146151 PMC4701435 26730600

[B22] Fulcher L. P. Scherer R. C. Melnykov A. V. Gateva V. Limes M. E. (2006). Negative coulomb damping, limit cycles, and self-oscillation of the vocal folds. Am. J. Phys. 74, 386–393. 10.1119/1.2173272

[B23] Giovanni A. Demolin D. Heim C. Triglia J.-M. (2000). Estimated subglottic pressure in normal and dysphonic subjects. Ann. Otology, Rhinology Laryngology 109, 500–504. 10.1177/000348940010900511 10823481

[B24] Gómez P. Schützenberger A. Kniesburges S. Bohr C. Döllinger M. (2018). Physical parameter estimation from porcine *ex vivo* vocal fold dynamics in an inverse problem framework. Biomechanics Model. Mechanobiol. 17, 777–792. 10.1007/s10237-017-0992-5 29230589

[B25] Gómez P. Schützenberger A. Semmler M. Döllinger M. (2019). Laryngeal pressure estimation with a recurrent neural network. IEEE J. Transl. Eng. Health Med. 7, 2000111–11. 10.1109/JTEHM.2018.2886021 30680252 PMC6331197

[B26] Gray S. D. Alipour F. Titze I. R. Hammond T. H. (2000). Biomechanical and histologic observations of vocal fold fibrous proteins. Ann. Otology, Rhinology Laryngology 109, 77–85. 10.1177/000348940010900115 10651418

[B27] Hochreiter S. Schmidhuber J. (1997). Long short-term memory. Neural Comput. 9, 1735–1780. 10.1162/neco.1997.9.8.1735 9377276

[B28] Ibarra E. J. Parra J. A. Alzamendi G. A. Cortés J. P. Espinoza V. M. Mehta D. D. (2021). Estimation of subglottal pressure, vocal fold collision pressure, and intrinsic laryngeal muscle activation from neck-surface vibration using a neural network framework and a voice production model. Front. Physiology 12, 732244. 10.3389/fphys.2021.732244 PMC8440844 34539451

[B29] Inwald E. C. Döllinger M. Schuster M. Eysholdt U. Bohr C. (2011). Multiparametric analysis of vocal fold vibrations in healthy and disordered voices in high-speed imaging. J. voice official J. Voice Found. 25 (5), 576–590. 10.1016/j.jvoice.2010.04.004 20728308

[B30] Ishizaka K. Flanagan J. L. (1972). Synthesis of voiced sounds from a two-mass model of the vocal cords. Bell Syst. Tech. J. 51, 1233–1268. 10.1002/j.1538-7305.1972.tb02651.x

[B31] Ketelslagers K. Bodt M. S. D. Wuyts F. L. de Heyning P. V. (2007). Relevance of subglottic pressure in normal and dysphonic subjects. Eur. Archives Oto-Rhino-Laryngology 264, 519–523. 10.1007/s00405-006-0212-x 17146639

[B32] Kingma D. P. Ba J. (2014). Adam: a method for stochastic optimization, 6980. CoRR abs/1412.

[B33] Kist A. M. Gómez P. Dubrovskiy D. Schlegel P. Kunduk M. Echternach M. (2021). A deep learning enhanced novel software tool for laryngeal dynamics analysis. J. speech, Lang. Hear. Res. JSLHR 64 (6), 1889–1903. 10.1044/2021_JSLHR-20-00498 34000199

[B34] Kunduk M. Doellinger M. Mcwhorter A. J. Lohscheller J. (2010). Assessment of the variability of vocal fold dynamics within and between recordings with high-speed imaging and by phonovibrogram. Laryngoscope 120, 981–987. 10.1002/lary.20832 20422695

[B35] LeCun Y. Bottou L. Bengio Y. Haffner P. (1998). Gradient-based learning applied to document recognition. Proc. IEEE 86, 2278–2324. 10.1109/5.726791

[B36] Lee Y.-S. (1995). Graphical demonstration of an optimality property of the median. Am. Statistician 49, 369–372. 10.2307/2684577

[B37] Lohscheller J. Švec J. G. Döllinger M. (2013). Vocal fold vibration amplitude, open quotient, speed quotient and their variability along glottal length: kymographic data from normal subjects. Logop. Phoniatr. Vocology 38, 182–192. 10.3109/14015439.2012.731083 23173880

[B38] Long W. Wu T. Liang X. Xu S. (2019). Solving high-dimensional global optimization problems using an improved sine cosine algorithm. Expert Syst. Appl. 123, 108–126. 10.1016/j.eswa.2018.11.032

[B39] Lucero J. C. Lourenço K. G. Hermant N. Hirtum A. V. Pelorson X. (2012). Effect of source-tract acoustical coupling on the oscillation onset of the vocal folds. J. Acoust. Soc. Am. 132 (1), 403–411. 10.1121/1.4728170 22779487

[B40] Moore J. Thibeault S. L. (2012). Insights into the role of elastin in vocal fold health and disease. J. voice official J. Voice Found. 26 (3), 269–275. 10.1016/j.jvoice.2011.05.003 PMC3190022 21708449

[B41] Nelsen R. B. (2006). An introduction to copulas. Springer.

[B42] Nerriere É. Vercambre M.-N. Gilbert F. Kovess-Masfety V. (2009). Voice disorders and mental health in teachers: a cross-sectional nationwide study. BMC Public Health 9, 370. 10.1186/1471-2458-9-370 19799781 PMC2762990

[B43] Nielsen M. A. (2015). Neural networks and deep learning, 25. San Francisco, CA, USA: Determination press.

[B44] Pascanu R. Mikolov T. Bengio Y. (2012). “On the difficulty of training recurrent neural networks,” in International Conference on Machine Learning.

[B45] Paszke A. Gross S. Massa F. Lerer A. Bradbury J. Chanan G. (2019). Pytorch: an imperative style, high-performance deep learning library. Adv. Neural Inf. Process. Syst. 32. Curran Associates, Inc., 8024–8035. 10.5555/3454287.3455008

[B46] Rumelhart D. E. Hinton G. E. Williams R. J. (1986). Learning representations by back-propagating errors. Nature 323, 533–536. 10.1038/323533a0

[B47] Schutzenberger A. Kunduk M. Döllinger M. Alexiou C. Dubrovskiy D. Semmler M. (2016). Laryngeal high-speed videoendoscopy: sensitivity of objective parameters towards recording frame rate. BioMed Res. Int. 2016, 4575437. 10.1155/2016/4575437 27990428 PMC5136634

[B48] Schwarz R. Döllinger M. Wurzbacher T. Eysholdt U. Lohscheller J. (2008). Spatio-temporal quantification of vocal fold vibrations using high-speed videoendoscopy and a biomechanical model. J. Acoust. Soc. Am. 123 (5), 2717–2732. 10.1121/1.2902167 18529190

[B49] Schwarz R. Hoppe H. U. Schuster M. Wurzbacher T. Eysholdt U. Lohscheller J. (2006). Classification of unilateral vocal fold paralysis by endoscopic digital high-speed recordings and inversion of a biomechanical model. IEEE Trans. Biomed. Eng. 53, 1099–1108. 10.1109/TBME.2006.873396 16761837

[B50] Semmler M. Berry D. A. Schützenberger A. Döllinger M. (2021). Fluid-structure-acoustic interactions in an *ex vivo* porcine phonation model. J. Acoust. Soc. Am. 149, 1657–1673. 10.1121/10.0003602 33765793 PMC7952141

[B51] Steinecke I. Herzel H. (1995). Bifurcations in an asymmetric vocal-fold model. J. Acoust. Soc. Am. 97 (3), 1874–1884. 10.1121/1.412061 7699169

[B52] Story B. H. Titze I. R. (1995). Voice simulation with a body-cover model of the vocal folds. J. Acoust. Soc. Am. 97 (2), 1249–1260. 10.1121/1.412234 7876446

[B53] Titze I. R. Riede T. Popolo P. S. (2008). Nonlinear source-filter coupling in phonation: vocal exercises. J. Acoust. Soc. Am. 123 (4), 1902–1915. 10.1121/1.2832339 18396999 PMC2677316

[B54] Van Rossum G. Drake F. L. (2009). Python 3 reference manual. Scotts Valley, CA: CreateSpace.

[B55] Wurzbacher T. Schwarz R. Döllinger M. Hoppe U. Eysholdt U. Lohscheller J. (2006). Model-based classification of nonstationary vocal fold vibrations. J. Acoust. Soc. Am. 120 (2), 1012–1027. 10.1121/1.2211550 16938988

[B56] Yang A. Lohscheller J. Berry D. A. Becker S. Eysholdt U. Voigt D. (2010). Biomechanical modeling of the three-dimensional aspects of human vocal fold dynamics. J. Acoust. Soc. Am. 127 (2), 1014–1031. 10.1121/1.3277165 20136223 PMC3137461

[B57] Zhang K. Siegmund T. Chan R. W. (2006a). A constitutive model of the human vocal fold cover for fundamental frequency regulation. J. Acoust. Soc. Am. 119 (2), 1050–1062. 10.1121/1.2159433 16521767

[B58] Zhang X. Gu L. Wei W. Wu D. Tao Z. Zhao H. (2018). Pathological voice source analysis system using a flow waveform-matched biomechanical model. Appl. Bionics Biomechanics 2018, 3158439. 10.1155/2018/3158439 PMC6051280 30057647

[B59] Zhang Z. (2020). Estimation of vocal fold physiology from voice acoustics using machine learning. J. Acoust. Soc. Am. 147 (3), EL264. 10.1121/10.0000927 32237804 PMC7075716

[B60] Zhang Z. Neubauer J. Berry D. A. (2006b). The influence of subglottal acoustics on laboratory models of phonation. J. Acoust. Soc. Am. 120 (3), 1558–1569. 10.1121/1.2225682 17004478

[B61] Zhou C. Sun C. Liu Z. Lau F. (2015). A c-lstm neural network for text classification. ArXiv abs/1511.08630.

