# Supplementary material for: Neural network-based estimation of biomechanical vocal fold parameters
Source: Front Physiol. 2024 Feb 21;15:1282574. doi: 10.3389/fphys.2024.1282574 (PMC10916882; doi:10.3389/fphys.2024.1282574)
Supplement: Supplementary file 1 [file DataSheet1.PDF]

Supplementary Material

1 EXPLICIT FORCE FORMULATION

The Six-Mass Model (6MM) forces (Schwarz et al., 2008) used in this work were computed as follows:

$$\boldsymbol{F}^{l}_{i,j,k} = F_{k_l,r_l}\left(\begin{pmatrix}\boldsymbol{x}_{i,j-1,k}-\boldsymbol{x}_{i,j,k}\\ l/4\\ 0\end{pmatrix}, \ell_l\right) - F_{k_l,r_l}\left(\begin{pmatrix}\boldsymbol{x}_{i,j,k}-\boldsymbol{x}_{i,j+1,k}\\ l/4\\ 0\end{pmatrix}, \ell_l\right) \tag{S1}$$

$$\boldsymbol{F}^{v}_{i,j,k} = F_{k_v,r_v}\left(\begin{pmatrix}\boldsymbol{x}_{i,j,k-1}-\boldsymbol{x}_{i,j,k}\\ 0\\ h\end{pmatrix}, \ell_v\right) - F_{k_v,r_v}\left(\begin{pmatrix}\boldsymbol{x}_{i,j,k}-\boldsymbol{x}_{i,j,k+1}\\ 0\\ h\end{pmatrix}, \ell_v\right) \tag{S2}$$

$$\boldsymbol{F}^{a}_{i,j,k} = F_{k_a,r_a}\left(\begin{pmatrix}\boldsymbol{x}_{i,j,k}-\boldsymbol{x}^{r}_{i,j,k}\\ 0\\ 0\end{pmatrix}, 0\right) \tag{S3}$$

$$-\boldsymbol{F}^{c}_{1,j,k} = \boldsymbol{F}^{c}_{2,j,k} = \Theta(\boldsymbol{x}_{1,j,k}-\boldsymbol{x}_{2,j,k})\, F_{k_c,r_c}\left(\begin{pmatrix}\boldsymbol{x}_{1,j,k}-\boldsymbol{x}_{2,j,k}\\ 0\\ 0\end{pmatrix}, 0\right) \tag{S4}$$

$$-\boldsymbol{F}^{d}_{1,j,1} = \boldsymbol{F}^{d}_{2,j,1} = P_S\left(1-\Theta(\boldsymbol{a}_{j,\min})\left(\frac{\boldsymbol{a}_{j,\min}}{\boldsymbol{a}_{j,1}}\right)^2\right)\Theta(\boldsymbol{a}_{j,1})\frac{lh}{3} \tag{S5}$$

where $\boldsymbol{x}_{i,0,k}$ denotes the fixed lateral posterior positions and $\boldsymbol{x}_{i,4,k}$ the anterior position respectively. Vocal Fold (VF) elongation is assumed to be l, the lower component height is assumed to be h. Area between masses is given as $\boldsymbol{a}_{j,k} := l/4 \cdot (\boldsymbol{x}_{2,j,k} - \boldsymbol{x}_{1,j,k})$, where $\boldsymbol{a}_{j,\min} := \min(\boldsymbol{a}_{j,1}, \boldsymbol{a}_{j,2})$. The force of a spring with free elongation ℓ and deflection $\boldsymbol{s}$ is denoted as $F_{k,r}(\boldsymbol{s}, \ell)$.

2 LIST OF ABBREVIATIONS

2MM	Two-Mass Model
6MM	Six-Mass Model
CDF	Cumulative Density Function
CNN	Convolutional Neural Network
CRNN	Convolutional Recurrent Neural Network
DOF	Degree-Of-Freedom
FC	Fully Connected
FFNN	Feedforward Neural Network
FPS	Frames Per Second
GRU	Gated Recurrent Unit
HSV	High-Speed Video endoscopy
LSTM	Long-Short Term Memory
MAE	Mean Absolute Error
MAPE	Mean Absolute Percentage Error
MMM	Multi-Mass Model
MSD	Mass-Sping-Damper
NN	Neural Network
ODE	Ordinary-Differential Equation
ReLU	Rectified Linear Unit
RNN	Recurrent Neural Network
RMSE	Root Mean Squared Error
RMSLE	Root Mean Squared Logarithmic Error
VF	Vocal Fold

REFERENCES

Schwarz, R., Döllinger, M., Wurzbacher, T., Eysholdt, U., and Lohscheller, J. (2008). Spatio-temporal quantification of vocal fold vibrations using high-speed videoendoscopy and a biomechanical model. *The Journal of the Acoustical Society of America* 123 5, 2717–32
